# Bayesian Networks Illustrate Genomic and Residual Trait Connections in Maize (*Zea mays* L.)

**DOI:** 10.1534/g3.117.044263

**Published:** 2017-06-21

**Authors:** Katrin Töpner, Guilherme J. M. Rosa, Daniel Gianola, Chris-Carolin Schön

**Affiliations:** * Plant Breeding, TUM School of Life Sciences Weihenstephan, Technical University of Munich, 85354 Freising, Germany; † Institute for Advanced Study, Technical University of Munich, 85748 Garching, Germany; ‡ Department of Animal Sciences, University of Wisconsin–Madison, Wisconsin 53706

**Keywords:** Bayesian network, structural equation model, multivariate mixed model, multiple-trait genome-enabled prediction, indirect selection

## Abstract

Relationships among traits were investigated on the genomic and residual levels using novel methodology. This included inference on these relationships via Bayesian networks and an assessment of the networks with structural equation models. The methodology employed three steps. First, a Bayesian multiple-trait Gaussian model was fitted to the data to decompose phenotypic values into their genomic and residual components. Second, genomic and residual network structures among traits were learned from estimates of these two components. Network learning was performed using six different algorithmic settings for comparison, of which two were score-based and four were constraint-based approaches. Third, structural equation model analyses ranked the networks in terms of goodness of fit and predictive ability, and compared them with the standard multiple-trait fully recursive network. The methodology was applied to experimental data representing the European heterotic maize pools Dent and Flint (*Zea mays* L.). Inferences on genomic and residual trait connections were depicted separately as directed acyclic graphs. These graphs provide information beyond mere pairwise genetic or residual associations between traits, illustrating for example conditional independencies and hinting at potential causal links among traits. Network analysis suggested some genetic correlations as potentially spurious. Genomic and residual networks were compared between Dent and Flint.

Selection exploits genetic properties of a population by passing favorable alleles onto subsequent generations. In breeding of animals and plants, it is crucial to distinguish between genetic (heritable) contributions and environmental factors influencing traits, and the decomposition of phenotypic variances and covariances between traits into their genetic and environmental components has been under investigation for decades (*e.g.*, Fisher 1918; Hazel 1943; Falconer 1952; Robertson 1959; Searle 1961; Roff 1995; Lynch and Walsh 1998). Identifying genetic relationships between traits is an important research focus; an example is a study of genetic correlations among 16 quantitative traits of maize (Malik *et al.* 2005). Insights into the connections between developmental or phenological traits with target traits such as stress resistance might assist breeding decisions. While such relationships may be antagonistic when two target traits affect each other negatively, they can be useful in genome-enabled prediction of traits when a complex trait is influenced by a second trait that is easier to measure, assess, or breed for. In addition, it has been found that selection for traits with low heritability can be enhanced by joint modeling with genetically correlated traits. Several advantages of multiple-trait prediction models have been corroborated by experimental and simulation studies (*e.g.*, Jia and Jannink 2012; Pszczola *et al.* 2013; Guo *et al.* 2014; Jiang *et al.* 2015; Maier *et al.* 2015).

Discovering and understanding phenotypic relations among traits is therefore important in this context. Statistical methods have been developed that specify connections among traits as directed influences, in contrast to standard multiple-trait models where connections among traits are represented by unstructured covariance matrices. One effective approach, structural equation models (SEM), can describe systems of phenotypes connected via feedback or recursive relationships (Gianola and Sorensen 2004). SEM are regression models that allow for a structured dependence among variables, *e.g.*, by a structure matrix Λ. Elements of Λ represent effects of dependence and can be either freely varying, or the structure can be constrained by setting some entries to zero *a priori*. For a given structure Λ, the effect of one variable on another can be estimated using likelihood-based or Bayesian approaches. SEM are well established and many studies in the animal sciences have investigated causal relationships among traits using various SEM approaches and data sets, *e.g.*, body composition and bone density in mouse intercross populations (Li *et al.* 2006), calving traits in cattle (de Maturana *et al.* 2009, 2010), or gene expression in mouse data by incorporating genetic markers (Schadt *et al.* 2005; Aten *et al.* 2008). Methodology, applications, and potential advantages of SEM in prediction of traits were reviewed by Rosa *et al.* (2011) and Valente *et al.* (2013). One of the limitations of SEM is that connections between traits must be assumed known to be able to estimate their magnitude.

To investigate connections among traits, Bayesian-network (BN) learning methods can be used. BN are models representing the joint distribution of random variables (*e.g.*, traits) in terms of their conditional independencies. There are two main types of algorithms for learning a BN: constraint-based and score-based algorithms. The former uses a sequence of conditional independence tests to learn the network among variables, while the latter compares the fit of many (ideally all) possible networks to the empirical data using a score. BN have been used for many purposes in quantitative genetics, for example, to predict individual total egg production of European quails using earlier expressed phenotypic traits (Felipe *et al.* 2015) and to study linkage disequilibrium using single nucleotide polymorphism (SNP) markers (Morota *et al.* 2012). BN have also been employed in genome-assisted prediction of traits, with a performance that was at least as good as that of other methods such as genomic best linear unbiased prediction or elastic nets (Scutari *et al.* 2014). In addition, many studies have investigated connections among several traits via a BN analysis incorporating quantitative trait loci and phenotypic data (Neto *et al.* 2008, 2010; Winrow *et al.* 2009; Hageman *et al.* 2011; Wang and van Eeuwijk 2014; Peñagaricano *et al.* 2015). BN can also search for connections among SNPs and traits for feature selection, or in genome-wide association studies by finding the Markov blanket for one or several traits (Porth *et al.* 2013; Scutari *et al.* 2013). However, interpretation of BN connections as causal influences is a delicate issue. Methodology and issues in causal inference were reviewed by Rockman (2008) and go back to Pearl (2000). Within the scope of the present study, BN are seen as a hypothesis-generating tool with respect to the causal nature of the connections found.

Valente *et al.* (2010) investigated causal connections among phenotypic traits using an approach combining BN and SEM. They learned network structure from Markov chain Monte Carlo (MCMC) samples of the residual covariance matrix of a Bayesian multiple-trait model using the inductive causation (IC) algorithm. This was a preliminary step before fitting an SEM with the selected structure to estimate the magnitude of influences among traits. These authors illustrated the effectiveness of their approach with a simulation study.

Here, we also combine BN and SEM analysis to investigate several traits simultaneously, but with respect to their genomic and residual relationships: arguably, genomic connections among traits must be different from residual relationships, the latter mainly due to environmental causes. First, we learn genomic and residual networks using different BN algorithms. The learned networks may provide a deeper insight into relationships among traits than pairwise-association measures (such as correlations or covariances). Instead of using the IC algorithm to analyze MCMC samples of covariance matrices, we use predicted (fitted) genomic values and residuals from a multiple-trait model as input variables for structure-learning algorithms. This approach allows using available software for learning BN, is computationally easy and fast, and use of bootstrapping to assess uncertainty regarding the edges is straightforward. Using this strategy, we study the behavior of various BN algorithms on experimental data. Afterward, we compare and assess the BN algorithms by fitting an SEM to the structures learned from them.

The methodology is illustrated using a maize data set. Biological objectives of this case study are the investigation of connections among maize traits and the comparison of networks with respect to two underlying sources of information (genomic and residual) and two heterotic groups (Dent and Flint). Five well-characterized complex traits were studied: biomass yield, plant height, maturity, and male and female flowering time. With these, well-known connections can be reconstructed and new insights into potentially spurious trait connections are obtained. After illustrating trait connections with respect to their genomic and residual sources and identifying spurious connections, a clearer picture on which traits should be included in a multiple-trait prediction model or in an indirect selection process may be gained.

## Materials and Methods

### Material

The data contain a sample of phenotypes and genotypes representing two important European heterotic pools, Dent and Flint (Bauer *et al.* 2013; Lehermeier *et al.* 2014). In the Dent (Flint) panel, 10 (11) founder lines were crossed to a common central Dent (Flint) line. Derived double haploid (DH) lines were evaluated as testcrosses with the central line from the opposite pool.

In both panels, five phenotypic traits were recorded: biomass dry matter yield (DMY) (decitons per hectare), biomass dry matter content (DMC) (percentage), plant height (PH) (centimeters), days to tasseling (DtTAS) (days), and days to silking (DtSILK) (days). Phenotypic values are available as adjusted means across locations and replications. The adjusted means were mean-centered in each DH population, as we focused on allelic rather than on population effects. All traits were standardized to unit variance based on their sample variance.

All DH lines of both panels were genotyped with the Illumina MaizeSNP50 BeadChip containing 56,110 SNP markers (Ganal *et al.* 2011). After imputation and SNP quality control, 34,116 high-quality SNPs remained in the Dent and Flint panels. Only markers that were polymorphic within a panel were considered, which were 32,801 (30,801) SNPs in the Dent (Flint) panel. A total of 831 (805) Dent (Flint) DH lines were used for analyses.

### Methods

The methodology includes a genetic and a residual structure that are expected to be different. The rationale behind this assumption is that genetic connections among traits do not necessarily resemble the residual ones, and vice versa; typically, genetic and residual factors are assumed to have independent distributions, reflecting control by disjoint factors.

Exploring these connections among traits includes the following steps: (1) fitting a Bayesian multiple-trait Gaussian model (MTM) to all five traits to obtain posterior means of genomic values and of model residuals for each panel, and transforming the predicted genomic values to meet assumptions on sample independence required by a BN analysis; (2) applying the BN analysis to the residual and transformed genomic trait components; and (3) assessing the quality of the inferred structures by a structure-comparative SEM analysis.

#### MTM:

We fitted a Bayesian multivariate Gaussian model to the d=5 traits and n=831 (n=805) genotypes within the Dent (Flint) panel to separate genomic values from random residual contributions to the phenotypic values, and to estimate the genomic and residual correlations between traits. The MTM model can be expressed as

$$y=\mu ⊗1_{n}+u+e,$$ (1)

where y, u, and e are (*nd* × 1)-dimensional column vectors of scaled adjusted means, genomic values, and residuals sorted by trait and genotype within trait, respectively; $\mu ⊗1_{n}$ is a Kronecker product of a (*d* × 1)-dimensional intercept vector μ and an (*n* × 1)-dimensional vector of ones. Since phenotypes y are mean centered, only trait-specific intercepts are included in the model as fixed effects represented by the d coefficients in μ.

The joint distribution of u and e was assumed to be multivariate normal with the following specification:

$$\left(\begin{array}{c} u\\ e \end{array}\right)∼N_{2dn}\left(\left(\begin{array}{c} 0\\ 0 \end{array}\right),\left(\begin{array}{cc} G⊗K & 0\\ 0 & R⊗I_{n\times n} \end{array}\right)\right),$$ (2)

where K is an (*n* × *n*)-dimensional realized-kinship matrix estimated using simple matching coefficients (Sneath and Sokal 1973). G and R are (*d* × *d*)-dimensional covariance matrices containing the genomic and residual variance and covariance components, and the (*n* × *n*)-dimensional identity matrix $I_{n\times n}$ represents the independence of residuals among genotypes.

Flat priors were assigned to the elements of the intercept vector μ. The covariance matrices G and R were assumed to follow *a priori* independent inverse-Wishart distributions with specific degrees of freedom ν and scale matrix Σ, regarded as hyper-parameters, *i.e.*,

$$G|{\text{Σ}}_{G},\nu_{G}\;∼\;W^{-1}\left({\text{Σ}}_{G},\nu_{G}\right)$$ (3)

and

$$R|{\text{Σ}}_{R},\nu_{R}\;∼\;W^{-1}\left({\text{Σ}}_{R},\nu_{R}\right).$$ (4)

The hyper-parameters ν and Σ were chosen such that: (1) they implied relatively uninformative prior distributions of matrices G and R, (2) the prior mode of genomic and residual trait variances was 0.5 for each trait, and (3) the choice of hyper-parameters needed to ensure that there exists an analog choice of hyper-parameters for a scaled-inverse $\chi^{2}$ prior defined later in the SEM models for trait variance components. Considering (1), (2), and (3), we set $\nu_{G}=\nu_{R}=8$ and ${\text{Σ}}_{G}={\text{Σ}}_{R}=7\cdot I_{d\times d},$ with ν and Σ in accordance with the parametrization of the respective distributions in the R package *BGLR* and the *MTM* function of its multiple-trait extension.

An MCMC approach based on Gibbs sampling was used to explore posterior distributions. A burn-in of 30,000 MCMC samples was followed by an additional 300,000 MCMC samples. The MCMC samples were thinned with a factor of two, resulting in 150,000 MCMC samples for inference. Posterior means were then calculated for μ, u, e, G, and R for the Dent and Flint panels separately. Genomic and residual trait correlations and their SDs were estimated from samples of the posterior distributions of G and R.

Subsequent BN analyses concentrated on $\hat{u}$ and $\hat{e}$ separately, the posterior mean estimates of u and e, respectively. Traditionally in quantitative genetics and plant breeding, phenotypic variation is decomposed into genetic and environmental random contributions; genotype-by-environment interaction can confound both of these contributions. In experimental data from plant breeding, phenotypic data points are usually averaged across locations representing different environmental conditions, and every location contains replications of experiments. This approach reduces noise from environmental effects and also effectively decreases the influence of genotype-by-environment effects in the data (Falconer and Mackay 1996). As a consequence, residuals estimated with model (1) from such data include, *e.g.*, nonadditive genetic effects and nonaccounted-for (micro-)environmental effects, such as nongenetic physiological and morphological trait dependencies. Random genomic values fitted with model (1) represent, *e.g.*, genetic connections among traits, induced *inter alia* by pleiotropy and linkage disequilibrium. Here, we take the fitted genomic values and residuals and investigate them with BN.

Predictive ability from a univariate Bayesian prediction model was derived for each trait with 10 replicates of a fivefold cross-validation to compare single-trait prediction with multiple-trait prediction. Prior assumptions were analogous to those of the MTM, that is, scaled-inverse $\chi^{2}$ distributions with four degrees of freedom and a scale parameter equal to three were employed for each of the genomic and the residual variances.

#### Transforming the genomic component to meet assumptions of the BN learning algorithms:

BN learning algorithms assume independent observations. In the MTM described above, independence of residuals between genotypes was assumed. In contrast, the genomic components in u were correlated between genotypes due to kinship (represented by the matrix K) in addition to being dependent within genotypes due to genomic trait covariance (represented by the matrix G). Before learning BN from the genomic component, a transformation was therefore applied to vector u such that the transformed vector u* was distributed as $N_{\mathrm{dn}}\left(0,G⊗I_{n\times n}\right),$ *i.e.*, elements of u* were independent between genotypes while still preserving genomic relationships among traits as encoded by G.

For this purpose, we decomposed the kinship matrix K into its Cholesky factors, $K=LL^{T},$ where L is an (*n* × *n*)-dimensional lower triangular matrix, and define a (*dn* × *dn*)-dimensional matrix $M=I_{d\times d}⊗L,$ so that $M^{-1}=I_{d\times d}⊗L^{-1}$ and $\left(I_{d\times d}⊗L\right)\left(I_{d\times d}⊗L^{-1}\right)=I_{\mathrm{dn}\times \mathrm{dn}}.$

For $\mathrm{u*}=M^{-1}u$ we have

$$\begin{array}{l} \mathrm{Var}\left(u^{*}\right)=\mathrm{Var}\left(M^{-1}u\right)=M^{-1}\mathrm{Var}\left(u\right){\left(M^{-1}\right)}^{T}=\\ M^{-1}\;\left(G⊗K\right){\left(M^{-1}\right)}^{T}=\left(I_{d\times d}⊗L^{-1}\right)\left(G⊗K\right){\left(I_{d\times d}⊗L^{-1}\right)}^{T}=G⊗I_{n\times n} \end{array}$$ (5)

because $L^{-1}K{\left(L^{-1}\right)}^{T}=I_{n\times n},$ which follows directly from the definition of the Cholesky factor. This transformation and the resulting covariance structure was used by Vázquez *et al.* (2010), but for a single-trait model.

For a standard Cholesky decomposition of K, a positive definite realized kinship matrix is needed. Realized kinship matrices are not guaranteed to be positive definite, in contrast to pedigree-based kinship matrices. However, adjustments to assure positive definiteness are available (Nazarian and Gezan 2016).

In subsequent BN analysis, U* and E denoted two (*n* × *d*)-dimensional matrices formed from the vectors $\mathrm{u*}=M^{-1}u$ and e by arranging genotypes and traits in rows and columns, respectively.

#### Learning genomic and residual BN:

A BN is a model that describes connections among random variables. It consists of two components: a statement about the joint distribution of these random variables, and its graphical representation as a directed acyclic graph (DAG) (Nagarajan *et al.* 2013). The statement about the joint distribution $f\left(V\right)=f\left(V_{1},\;V_{2},\;\ldots ,\;V_{K}\right)$ of the K random variables $V_{1},V_{2},\ldots ,\;V_{K}$ is that f(V) can be decomposed into a product of conditional distributions:

$$f\left(V\right)=f\left(V_{1},V_{2},\ldots ,V_{K}\right)=\prod_{k=1}^{K}f\left(V_{k}|pa\left(V_{k}\right)\right).$$ (6)

Above, $pa\left(V_{k}\right),$ the “parents” of $V_{k},$ denotes the subset of random variables in V on which $V_{k}$ depends. Accordingly, $V_{k}$ is then called the “child” of all elements in $pa\left(V_{k}\right).$ In the respective DAG, all random variables $V_{k}$ are represented as nodes, and arcs point from parents to their children. The joint distribution is also referred to as a global distribution and the conditional distributions are called local distributions. If the global and local distributions are normal and the variables are linked by linear constraints, the BN is a Gaussian BN.

**Figure 1:**
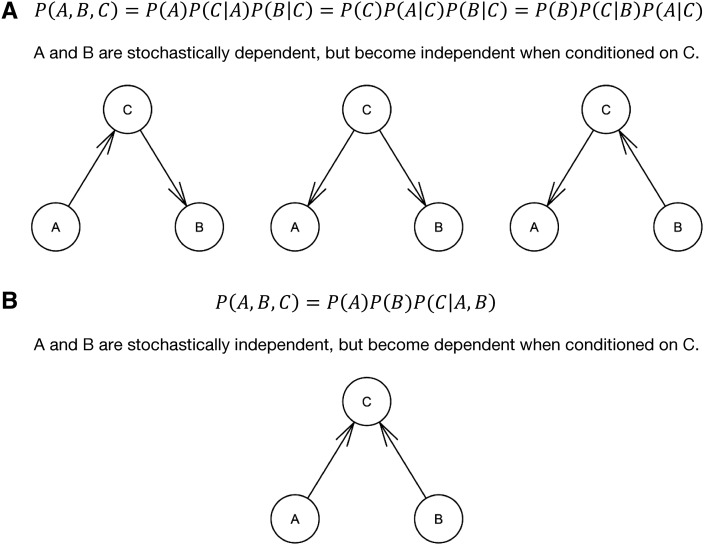
Structure learning: constraint-based algorithms search for trios of variables and test their relations with marginal and conditional independence tests. Thereby, they distinguish between the stochastic decomposition of the joint distribution of all variables as in (A) and the decomposition as in (B). These two decompositions can be represented in a DAG as shown. Whereas the directions are clear and unique in (B), the influences’ directions in (A) can form three different graphs. Which of these equivalent structures is most likely, one can only deduce from the context of other already tested decompositions in their neighborhood and from the rule that no cycles must be formed in the graph.

Here, the random variables investigated are the genomic and residual components of phenotypic traits. We chose a Gaussian BN as a consequence of assuming that quantitative traits, and particularly genomic values and residuals, follow a normal distribution. Many tests and scores for learning BN assume independence among the observations of the random variables. In our study, the realized random variables were the genomic values and the residuals from the MTM, of which the former had to be transformed as described above. So we searched for the decomposition of the following global distributions:

$$f\left(U^{*}\right)=f\left(U_{\cdot 1}^{*},U_{\cdot 2}^{*},\ldots ,U_{\cdot 5}^{*}\right)=\prod_{k=1}^{5}f\left(U_{\cdot k}^{*}|pa\left(U_{\cdot k}^{*}\right)\right)$$ (7)

and

$$f\left(E\right)=f\left(E_{\cdot 1},E_{\cdot 2},\ldots ,E_{\cdot 5}\right)=\prod_{k=1}^{5}f\left(E_{\cdot k}\text{|}pa\left(E_{\cdot k}\right)\right),$$ (8)

where the index “⋅k” denotes the *k*th column of the respective matrix. There are two types of algorithms to learn the structure of networks: constraint-based and score-based algorithms (Nagarajan *et al.* 2013). The constraint-based algorithms employ a series of conditional and marginal independence tests to infer potential connections and directions between each pair of variables. First, an undirected structure linking variables directly related to each other is constructed. Next, trios of variables where one is the outcome of the other two (“v-structures”) are sought. Last, the remaining connections are oriented whenever possible, such that neither cycles nor new v-structures are created. For illustration, core structures with three nodes are shown in Figure 1. In contrast, score-based approaches search through the space of possible networks (including direction of edges) and compare them by a goodness-of-fit score, returning the network with the highest score.

In this study, we chose the Grow-Shrink algorithm (GS) (Margaritis 2003) as an example of a constraint-based approach, and tabu-search (TABU) (Daly and Shen 2007) as an example of a score-based algorithm. For comparing the constraint-based with the score-based approach, these two algorithms are ideal when dealing with small networks of up to 10–15 traits. For larger networks, runtime-optimized network learning algorithms might be preferred.

The GS was combined with four different tests for marginal and conditional independence. We used the simple correlation coefficient with an exact Student’s *t*-test (GS 1) and with a Monte Carlo permutation test (GS 2) (Legendre 2000). Further, GS was combined with mutual information, an information-theoretic distance measure. It was investigated with an asymptotic $\chi^{2}$ test (GS 3) and with a Monte Carlo permutation test (GS 4) (Kullback 1959). Multiple testing issues in the context of a constraint-based algorithm have been addressed by Aliferis *et al.* (2010a,b). According to these authors, for a sample size of ∼1000, a significance level of α=0.05 resulted in a worst-case false positive rate of $6\times 10^{-4}$ for arc inclusion. Choosing α=0.01 in the more exhaustive GS, false positives should be avoided effectively with five variables (traits) and ∼800 genotypes.

The TABU was combined with two different scores: the Bayesian information criterion (BIC) (Rissanen 1978; Lam and Bacchus 1994) (TABU 1) and the Bayesian Gaussian equivalent score (BGE) (Geiger and Heckerman 1995) (TABU 2). The BGE is a Bayesian scoring metric for Gaussian BN with many favorable properties. *Inter alia*, BGE assigns the same scores to equivalent network structures (Chickering 1995). Equivalent network structures refer to the same decomposition of the global distribution with different edge directions (*e.g.*, part A of Figure 1).

In total, six (GS 1, GS 2, GS 3, GS 4, TABU 1, and TABU 2) different learning settings were applied to the genomic and residual components of model (1), respectively; all algorithms assuming a Gaussian BN. Regarding the constraint-based approaches, the number of permutations used for each of the permutation tests was 450 (slightly more than half of the genotypes in each of the two panels). Each of the six settings was run with 500 bootstrap samples and the significance level was set to 0.01 (as mentioned above). After structure learning from the bootstrap samples, we averaged over the 500 resulting networks as described in the R package *bnlearn* (Scutari 2010; Nagarajan *et al.* 2013). Significance of edges in the averaging process was assessed by an empirical test on the arcs’ strength (Scutari and Nagarajan 2011).

The structure learned by the BN needed to be translated into a structure matrix for SEM analysis. Thus, a (*d* × *d*)-dimensional matrix Λ was formed from each learned structure. Each arrow of the DAG pointing from a parent trait to a child trait becomes a freely varying coefficient in the structure matrix in the column of the parent and the row of the child. All other entries were set to zero *a priori*. The resulting structure matrices are lower triangular matrices, since loops are not allowed in a BN structure. The lower triangular form might not be produced by an arbitrary order of traits but can always be formed by rearranging the order of the traits by columns and rows; see Supplemental Material, Figure S1, for an example of how a DAG is transformed into a structure matrix. If a learned structure was an element of an equivalent class of several structures, only one representative of this equivalent class was translated into a structure matrix Λ and assessed in SEM analyses. Different SEM based on different Λ from the same equivalent class may lead to different sets of coefficients. However, they lead to the same deviance information criterion (DIC) or likelihood of the respective SEM.

Hereinafter, $\Lambda_{\hat{U}*}{}_{}$ denotes the structure matrix of a network learned from $\hat{U}*,$ and $\Lambda_{\hat{E}}$ denotes the structure matrix of a network learned from $\hat{E}.$

#### Assessing BN inference by SEM:

In the following paragraph, we present how the structures found in the BN analysis translate into SEM; doing so also clarifies how MTM and SEM are related. Genomic or residual trait dependence can be described by the recursive linear regression models

$$\mathrm{u*}=\left(\Lambda_{\mathrm{U*}}⊗I_{n\times n}\right)\mathrm{u*}+p_{\mathrm{u*}}$$ (9)

and

$$e=\left(\Lambda_{E}⊗I_{n\times n}\right)e+p_{e},$$ (10)

where $\Lambda_{\mathrm{U*}}$ and $\Lambda_{E}$ denote the (*d* × *d*)-dimensional genomic and residual structure matrices and $p_{\mathrm{u*}}$ $\left(p_{e}\right)$ are dn independent and identically normal-distributed residuals. Independence among genotypes in $p_{\mathrm{u*}}$ follows from the transformation of u into u*; elements of $p_{e}$ are independent since e are independent among genotypes by the model assumptions of MTM. If the genomic and residual structures in Equations 9 and 10 explain all genomic and residual trait covariances (which true structures ideally do), then $p_{\mathrm{u*}}$ and $p_{e}$ are also independent between traits.

For genotype i, the models are

$$u_{i}^{*}=\Lambda_{\mathrm{U*}}u_{i}^{*}+\left(p_{\mathrm{u*}}\right){}_{i}$$ (11)

and

$$e_{i}=\Lambda_{E}e_{i}+(p_{e}){}_{i}.$$ (12)

Equations 11 and 12 can be rearranged into

$$u_{i}^{*}={\left(I_{d\times d}-\Lambda_{\mathrm{U*}}\right)}^{-1}\left(p_{\mathrm{u*}}\right){}_{i}$$ (13)

and

$$e_{i}={\left(I_{d\times d}-\Lambda_{E}\right)}^{-1}(p_{e}){}_{i},$$ (14)

which imply that

$$\mathrm{Var}\left(u_{i}^{*}\right)=\mathrm{Var}\left[{\left(I_{d\times d}-\Lambda_{\mathrm{U*}}\right)}^{-1}\left(p_{\mathrm{u*}}\right){}_{i}\right]={\left(I_{d\times d}-\Lambda_{\mathrm{U*}}\right)}^{-1}\mathrm{Var}\left(\left(p_{\mathrm{u*}}\right){}_{i}\right){\left[{\left(I_{d\times d}-\Lambda_{\mathrm{U*}}\right)}^{-1}\right]}^{T}$$ (15)

and

$$\mathrm{Var}\left(e_{i}\right)=\mathrm{Var}\left[{\left(I_{d\times d}-\Lambda_{E}\right)}^{-1}\left(p_{e}\right){}_{i}\right]={\left(I_{d\times d}-\Lambda_{E}\right)}^{-1}\mathrm{Var}\left(\left(p_{e}\right){}_{i}\right){\left[{\left(I_{d\times d}-\Lambda_{E}\right)}^{-1}\right]}^{T},$$ (16)

where $\mathrm{Var}\left(\left(p_{\mathrm{u*}}\right){}_{i}\right)$ and $\mathrm{Var}\left(\left(p_{\mathrm{u*}}\right){}_{i}\right)$ are unknown diagonal matrices. Let $\Psi_{u}=\mathrm{Var}\left(\left(p_{\mathrm{u*}}\right){}_{i}\right)$ and $\Psi_{e}=\mathrm{Var}\left(\left(p_{\mathrm{u*}}\right){}_{i}\right)$ for all i, *i.e.*, the model is homoscedastic. Note that $\mathrm{Var}\left(u_{i}^{*}\right)=\mathrm{Var}\left(u_{i}\right)$ when considering individual genotypes. Assuming that both genomic and residual variances of traits are the same for all genotypes, the genomic and residual covariance matrices G and R can be replaced by

$$\mathrm{G*}=\mathrm{Var}\left(u_{i}^{*}\right)=\mathrm{Var}\left(u_{i}\right)={\left(I_{d\times d}-\Lambda_{U^{*}}\right)}^{-1}\Psi_{u}{\left[{\left(I_{d\times d}-\Lambda_{U^{*}}\right)}^{-1}\right]}^{T}$$ (17)

for all *i* and

$$\mathrm{R*}=\mathrm{Var}\left(e_{i}\right)={\left(I_{d\times d}-\;\Lambda_{E}\right)}^{-1}\Psi_{e}{\left[{\left(I_{d\times d}-\Lambda_{E}\right)}^{-1}\right]}^{T}$$ (18)

for all *i*. Since the true structure matrices $\Lambda_{U*}$ and $\Lambda_{E}$ are unknown, the inferred structures from the BN, $\Lambda_{\hat{U}*}$ and $\Lambda_{\hat{E}},$ are used instead.

Then, the MTM can be reformulated as the following SEM in its reduced form (Gianola and Sorensen 2004; Valente *et al.* 2010):

$$y=\mu ⊗1_{n}+u+e,$$ (19)

where $\left(\begin{array}{c} u\\ e \end{array}\right)∼N_{2dn}\left(\left(\begin{array}{c} 0\\ 0 \end{array}\right),\left(\begin{array}{cc} \mathrm{G*}⊗K & 0\\ 0 & \mathrm{R*}⊗I_{n\times n} \end{array}\right)\right).$

The Bayesian Gibbs sampling implementation is as for a MTM, but, additionally, G* (R*) is sampled from its posterior distribution based on Equation 17 (Equation 18). The structures of $\Lambda_{\hat{U}*}$ and $\Lambda_{\hat{E}}$ affect whether or not the use of G* and R* instead of G and R induces a reduction in the number of parameters. For example, G* and G (R* and R) have the same number of nonnull parameters if the structure is fully recursive.

In our Bayesian model, the diagonal elements of matrices ${\text{Ψ}}_{u}$ and ${\text{Ψ}}_{e}$ were assigned independent scaled-inverse $\chi^{2}$ prior distributions with four degrees of freedom and scale of three. As mentioned above, this choice of hyper-parameters yields the same prior distribution as in the MTM with respect to the diagonal elements of the covariance matrices. Also, the trait variances’ prior mode is again 0.5 and the priors are relatively uninformative. The nonnull coefficients of $\Lambda_{\hat{U}*}$ and $\Lambda_{\hat{E}}$ were assigned independent normal distributions with null mean and variance of 6.3 and 0.07, respectively.

The prior distribution’s variance for the nonnull coefficients of $\Lambda_{\hat{U}*}$ and $\Lambda_{\hat{E}}$ was chosen to align with the estimated genomic (residual) covariance components in the MTM, which lay in the interval between −3.7 (−0.13) and 5.0 (0.52). When a normal prior distribution with null mean is assumed for the SEM structure matrices’ coefficients, then 5.0 (0.52) lies within two times its SD when its variance (a hyper-parameter) is chosen to be larger than ${\left(5/2\right)}^{2}=6.25$ $\left[{\left(0.52/2\right)}^{2}=0.0676\right].$

Predictive ability and goodness of fit of the MTM *vs.* the SEM were used to assess if the structures found in BN analysis were a better representation of trait connections than a fully recursive structure. We employed the DIC (Spiegelhalter *et al.* 2002), the marginal likelihood, and the predictive ability from 10 replicates of a fivefold cross-validation. The same 50 training- and test-set combinations were used for all models, including the single-trait model. Predictive ability was measured as the correlation between the adjusted means y and their predicted values $\hat{y}$ derived from the MTM or SEM. We fitted SEM with both or only one structured component, *i.e.*, SEM with G* and R*, G and R*, or G* and R.

### Data availability

Phenotypic data are available from file S1 of Lehermeier *et al.* (2014) at http://www.genetics.org/content/suppl/2014/09/17/198.1.3.DC1. Genotypic data were deposited in a previous project (Bauer *et al.* 2013) at National Center for Biotechnology Information Gene Expression Omnibus as data set GSE50558 (http://www.ncbi.nlm.nih.gov/geo/query/acc.cgi?acc=GSE50558). Analyses were performed with the statistical programming tool R (R Core Team 2014). Single-trait prediction was implemented with the *BLR* function (de los Campos and Pérez-Rodríguez 2012). Structure learning, MTM, and SEM analyses were implemented using the R packages *bnlearn* (Scutari 2010; Nagarajan *et al.* 2013) and an extension of the *BGLR* package (de los Campos and Pérez-Rodríguez 2014; Lehermeier *et al.* 2015) which is available on github (https://github.com/QuantGen/MTM).

## Results

### Trait correlations

Phenotypic, genomic, and residual correlations between the five traits were estimated with the MTM for the Flint and Dent panels separately (Table 1). In both panels, there was a strong positive genomic correlation between flowering traits DtTAS and DtSILK, and between the yield-related traits DMY and PH. Genomic correlations were positive for trait combinations not including DMC, whereas DMC was negatively correlated with all other traits. The magnitude of genomic correlations differed between the two panels; for example, the genomic correlation between DMC and DMY was −0.29 in the Dent panel, and −0.64 in the Flint panel. Posterior SDs of correlation estimates varied between 0.01 and 0.10. Single-trait predictive abilities (Table 1) were high and similar to those found in Lehermeier *et al.* (2014), where a detailed single-trait analysis of the phenotypic and genotypic data can be found.

**Table 1:** Phenotypic, genomic, and residual correlations for five traits in Dent (lower triangular) and Flint (upper triangular) with posterior SDs in parentheses where appropriate as well as single-trait predictive abilities

Dent\Flint	DMY	DMC	PH	DtTAS	DtSILK
Phenotypic correlations					
DMY		−0.36	0.68	0.60	0.56
DMC	−0.13		−0.50	−0.59	−0.64
PH	0.59	−0.27		0.66	0.66
DtTAS	0.39	−0.51	0.46		0.91
DtSILK	0.32	−0.57	0.45	0.80	
Genomic correlations					
DMY		−0.64 (0.07)	0.82 (0.04)	0.75 (0.05)	0.73 (0.05)
DMC	−0.29 (0.10)		−0.65 (0.06)	−0.71 (0.05)	−0.77 (0.04)
PH	0.79 (0.04)	−0.27 (0.08)		0.76 (0.04)	0.75 (0.04)
DtTAS	0.60 (0.07)	−0.66 (0.06)	0.60 (0.06)		0.95 (0.01)
DtSILK	0.46 (0.08)	−0.67 (0.06)	0.52 (0.07)	0.88 (0.02)	
Residual correlations					
DMY		0.14 (0.05)	0.41 (0.04)	0.19 (0.05)	0.14 (0.06)
DMC	0.06 (0.05)		−0.10 (0.06)	−0.26 (0.05)	−0.25 (0.05)
PH	0.33 (0.05)	−0.22 (0.05)		0.35 (0.05)	0.36 (0.05)
DtTAS	0.20 (0.05)	−0.26 (0.05)	0.25 (0.05)		0.68 (0.03)
DtSILK	0.17 (0.05)	−0.35 (0.05)	0.34 (0.05)	0.63 (0.03)	
Single-trait predictive abilities*^a^*					
Flint	0.63 (0.04)	0.66 (0.05)	0.69 (0.04)	0.74 (0.04)	0.76 (0.04)
Dent	0.51 (0.05)	0.64 (0.04)	0.69 (0.04)	0.61 (0.04)	0.68 (0.03)

Traits: biomass dry matter yield (DMY) (dt/ha), biomass dry matter content (DMC) (%), plant height (PH) (cm), days to tasseling (DtTAS) (days), days to silking (DtSILK) (days).

a Average of 10 random fivefold cross-validations.

### Transformation of the genomic component

**Figure 2:**
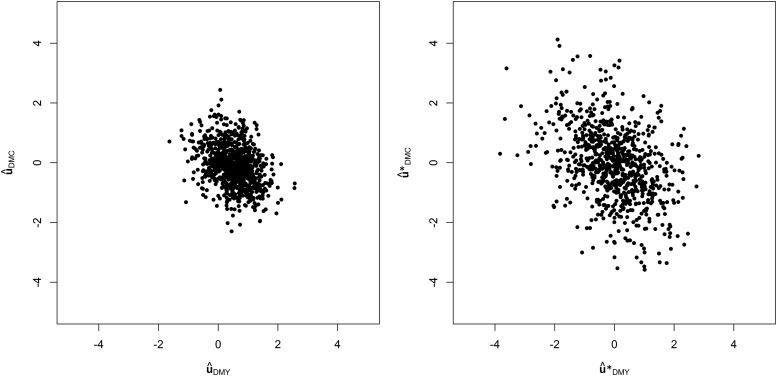
Transformation of the genomic component: relationships between estimated genomic values of DMY ${\hat{u}}_{DMY}$ and DMC ${\hat{u}}_{DMC}$ from the Dent panel and their counterparts $({\hat{u}*}_{DMY}^{*}$ and ${\hat{u}*}_{DMC}^{*})$ after transformation.

As the genotypic values in the MTM were correlated between genotypes due to kinship, estimates were transformed to meet the model assumptions of the BN algorithms. The transformation modified the relationship between estimated genotypic values as expected (as an example, see Figure 2).

### BN

BN inference was sensitive to the choice of algorithm and test (for detailed results see Figure S2, Figure S3, Figure S4, and Figure S5). The inferred networks varied in up to two edges and/or three directions. In general, score-based algorithms tended to choose more edges than constraint-based algorithms, and the networks of residual components were sparser than those of genomic components. Within the constraint-based or score-based approaches, inference was more consistent than between them.

BN inference of residual components was very similar in the Dent and Flint panels, irrespective of the algorithmic settings (Figure S3 and Figure S5). Connections that appeared in both panels were those between the flowering traits, from DtSILK to PH, from DMY to DMC, and between DMY and PH. Specific to the Flint panel was the edge from DtTAS to DMC, while the Dent panel showed a corresponding connection from DtSILK to DMC. An additional edge in the Dent panel not detected in the Flint panel was between DMC and PH. One network in the Flint panel showed an edge from PH to DtTAS, but it was only supported by 56% of the bootstrap samples in the respective network algorithm.

BN inference of genomic components was more variable than that of residual components within and across panels. In the Flint panel, algorithms recognized edges from DtSILK to DtTAS, from DtTAS to PH, from DtSILK to DMC; and between DMY and DMC, between DMY and DtTAS, between DMY and PH, and between DMC and DtTAS. In the Dent panel, only the connection between DMC and DMY was never inferred by any algorithm. A fully recursive network (*i.e.*, including all possible arcs) was never inferred, neither on the genomic nor on the residual components.

### Network assessment by SEM analysis

For assessing the structures inferred by the different BN algorithms, we integrated them into SEM, and compared them with MTM using goodness-of-fit criteria and predictive ability (Table 2, Table 3, Table S1, and Table S2). When two or more BN algorithms inferred the same network for the same component (genomic or residual), SEM analysis was only performed once for this structure. In the SEM, genomic and residual networks were assessed separately (G and R* or G* and R) (Table 2 and Table S1) and jointly (G* and R*) (Table 3 and Table S2).

**Table 2:** Single-structure evaluation: goodness of fit for the MTM and for SEM including a genomic $\left(\Lambda_{\hat{U}*}\right)$ or residual $\left(\Lambda_{\hat{E}}\right)$ trait structure denoted by the BN algorithm it originates from

BN Giving $\Lambda_{\hat{U}*}$	BN Giving $\Lambda_{\hat{E}}$	DIC*^a^*	p_D_*^b^*	logL*^c^*	No. Connections*^d^*	No. Nonnull Parameters*^e^*
Dent						
—	GS 3	−87.0	+61.6	+66.0	10 + 6 = 16	15 + 13 = 28
—	GS 1, 2, 4	−78.5	+65.7	+72.1	10 + 5 = 15	15 + 12 = 27
—	TABU 1, 2	−62.7	+45.1	+23.3	10 + 6 = 16	15 + 15 = 30
TABU 1	—	−0.7	−12.6	+12.1	8 + 10 = 18	15 + 15 = 30
—	—	0	1024.9*^f^*	0	20	30
TABU 2	—	+1.0	−14.1	−7.3	9 + 10 = 19	15 + 15 = 30
GS 1, 2, 3, 4	—	+57.0	−16.9	−32.4	7 + 10 = 17	15 + 15 = 30
Flint						
—	TABU 1	−124.1	+64.0	+105.2	10 + 5 = 15	15 + 13 = 28
—	GS 1, 2, 3, 4	−122.3	+63.3	+126.3	10 + 5 = 15	15 + 13 = 28
—	TABU 2	−117.7	+61.0	+131.1	10 + 6 = 16	15 + 14 = 29
—	—	0	1086.0*^f^*	0	20	30
TABU 1, 2	—	+35.8	+23.1	−3.2	7 + 10 = 17	14 + 15 = 29
GS1, 2, 3, 4	—	+72.1	+4.2	−57.1	6 + 10 = 16	12 + 15 = 27

For notation of BN algorithms see *Material and Methods*, *Learning genomic and residual BN*.

a Deviance information criterion: DIC of SEM minus DIC of MTM.

b Effective number of parameters: p_D_ of SEM minus p_D_ of MTM.

c Logarithm of Bayesian marginal likelihood: logL of SEM minus logL of MTM.

d Sum of connections in the networks: genomic plus residual.

e Sum of nonnull parameters in the covariance matrices: genomic plus residual.

f Absolute value of p_D._

**Table 3:** Double-structure evaluation: goodness of fit for MTM and for SEM including both genomic $\left(\Lambda_{\hat{U}*}\right)$ and residual $\left(\Lambda_{\hat{E}}\right)$ trait structure denoted by the BN algorithms they originate from

BN Giving $\Lambda_{\hat{U}*}$	BN Giving $\Lambda_{\hat{E}}$	DIC*^a^*	p_D_*^b^*	logL*^c^*	No. Connections*^d^*	No. Nonnull Parameters*^e^*
Dent						
TABU 1	GS 3	−87.0	+47.6	+63.8	8 + 6 = 14	15 + 13 = 28
TABU 2	GS 3	−86.0	+47.4	+66.1	9 + 6 = 15	15 + 13 = 30
TABU 1	GS 1, 2, 4	−75.1	+48.0	+35.5	8 + 5 = 13	15 + 12 = 27
TABU 2	GS 1, 2, 4	−74.4	+47.8	+52.7	9 + 5 = 14	15 + 12 = 27
TABU 1	TABU 1, 2	−64.3	+34.6	+66.0	8 + 6 = 14	15 + 15 = 30
TABU 2	TABU 1, 2	−63.9	+34.5	+54.1	8 + 6 = 14	15 + 15 = 30
GS 1, 2, 3, 4	GS 3	−31.2	+47.0	+41.3	7 + 6 = 13	15 + 13 = 28
GS 1, 2, 3, 4	GS 1, 2, 4	−26.6	+51.3	+21.1	7 + 5 = 12	15 + 12 = 27
GS 1, 2, 3, 4	TABU 1, 2	−9.2	+33.2	+23.0	7 + 6 = 13	15 + 15 = 30
—	—	0	1024.9*^f^*	0	20	30
Flint						
TABU 1, 2	TABU 1	−17.2	+108.1	+52.0	7 + 5 = 12	14 + 13 = 27
GS 1, 2, 3, 4	TABU 2	−15.6	+77.7	+40.4	6 + 6 = 12	12 + 14 = 26
TABU 1, 2	GS 1, 2, 3, 4	−14.8	+105.5	+65.9	7 + 5 = 12	14 + 13 = 27
TABU 1, 2	TABU 2	−14.0	+103.5	+65.3	7 + 6 = 13	14 + 14 = 28
—	—	0	1086.0*^f^*	0	20	30
GS1, 2, 3, 4	TABU 1	+30.4	+76.5	+34.8	6 + 5 = 11	12 + 13 = 25
GS1, 2, 3, 4	GS 1, 2, 3, 4	+33.1	+74.2	+22.9	6 + 5 = 11	12 + 13 = 25

For notation of BN algorithms see *Material and Methods*, *Learning genomic and residual BN*.

a Deviance information criterion: DIC of SEM minus DIC of MTM.

b Effective number of parameters: p_D_ of SEM minus p_D_ of MTM.

c Logarithm of Bayesian marginal likelihood: logL of SEM minus logL of MTM.

d Sum of connections in the networks: genomic plus residual.

e Sum of nonnull parameters in the covariance matrices: genomic plus residual.

f Absolute value of p_D._

For illustration of the variable reduction attained by application of the structures to the residual or genomic components, the number of connections in the networks and the resulting number of nonnull entries in G* and R* were derived (Table 2 and Table 3). For each SEM, the sum of the number of connections in the networks and the sum of nonnull entries in the covariance matrices were split into the genomic and the residual contributions. In general, residual networks were sparser than genomic networks, and SEM on residual structures were more parsimonious in the Flint panel than in the Dent panel.

Goodness of fit was assessed with the DIC and with the logarithm of the Bayesian marginal likelihood (logL) (Table 2 and Table 3). logL evaluates the fit of the model to the data and prior assumptions, and DIC takes into account and penalizes the number of parameters in the model. Models were ranked according to their DIC, which differed from their logL ranking. In Dent (Flint), eight (four) SEM with both a structured residual and structured genomic component had a lower DIC than the MTM (Table 3). In Flint, no SEM with only a genomic structure had a lower DIC than the MTM (Table 2). In general, DIC and logL varied more in Flint than in Dent (Table 2 and Table 3).

### Predictive abilities of SEM

Predictive abilities and their SDs were derived from 10 replicates of a fivefold cross-validation with random sampling of training and test sets within each panel (Table S1 and Table S2). Averaged over traits and for each trait individually, predictive abilities were similar for all models (single-trait, MTM, and SEM) and differences between models were <1 SD and not significant. The magnitude of predictive-ability estimates in the single-structure SEM was consistent with predictive-ability estimates in the double-structure SEM in both panels. Since all SEM with a genomic structure in the Flint panel had higher DIC (Table 2) and slightly lower predictive ability estimates (Table S1) than the MTM, genomic structures might not have been identified correctly, which might also have affected the double-structure SEM.

### Goodness of fit of SEM and choice of best networks

**Figure 3:**
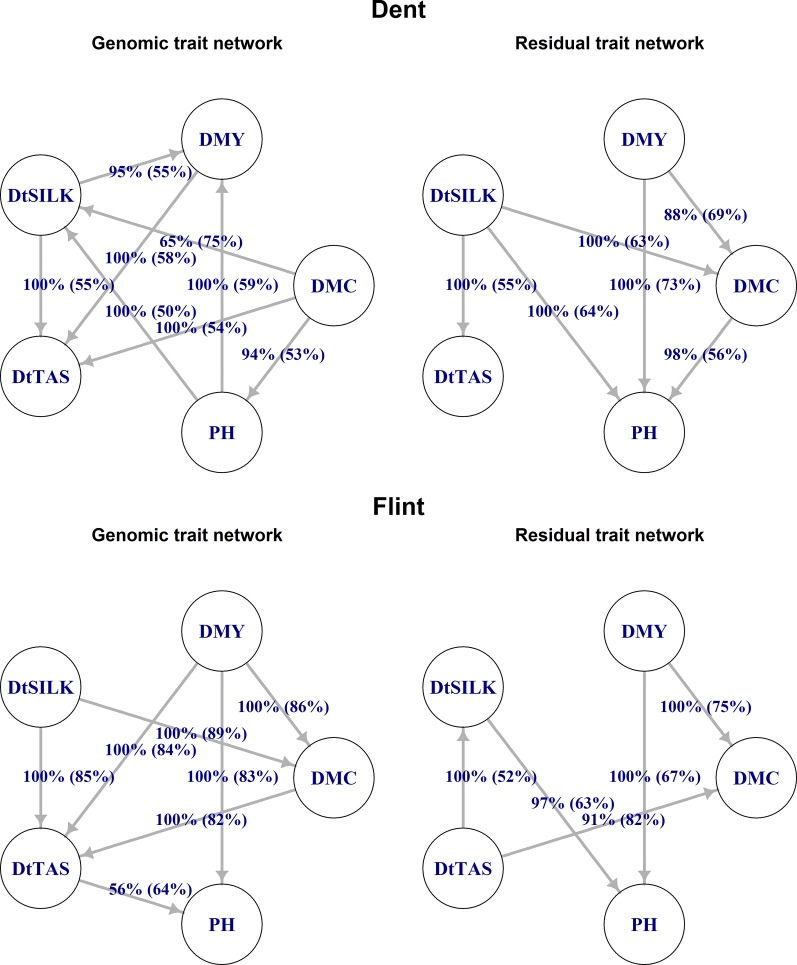
Trait networks for genomic values and residuals in Dent and Flint: graphs of the genomic component from the tabu-search algorithm using BIC as score. Structure learning was performed with 500 bootstrap samples. Graphs of the residuals from the GS algorithm with mutual information as test statistic, also with 500 bootstrap samples and a significance level of *α* = 0.01. Labels of edges indicate the proportion of bootstrap samples supporting the edge and (in parentheses) the proportion having the direction shown. Edges that were not significant in the averaging process due to a network-internal empirical test on the arc’s strength are not shown.

Considering all model performance criteria jointly, the best-performing networks in the SEM analysis are given in Figure 3. In the Dent panel, the SEM with the structure derived from the residuals with GS 3 and the SEM derived from the genomic component with TABU 1 performed best considering both the double-structure and single-structure settings. In the Flint panel, the SEM with the structure derived from the genomic component with TABU 1 and 2 had a better fit than the SEM with the structure derived from the genomic component with GS 1, 2, 3, and 4, although both had a higher DIC than the MTM. Regarding the residual structure in the Flint panel, we selected the structure derived with GS 1, 2, 3, and 4 because the SEM with the structure derived with TABU 2 had a higher DIC than the SEM with the structure derived with GS 1, 2, 3, and 4; and the SEM with the structure derived with TABU 1 had a considerably smaller logL than the SEM with the structure derived with GS 1, 2, 3, and 4. Consequently, Figure 3 shows the structures derived with TABU 1 for the genomic components and the structure derived with GS 3 for the residual components for both panels.

## Discussion

### Scope of methodology

Structures derived from BN algorithms conveyed more information than mere trait-association values from an MTM because many pairwise correlations are suggested to arise from indirect associations that are mediated by one or more variables. For example, the relatively strong genomic correlations between PH and DMC and between DtSILK and DMY in the Flint panel, and the relatively strong genomic correlation between DtSILK and DMC in the Dent panel, did not correspond to an edge in the genomic networks (Figure 3). As illustrated by Valente *et al.* (2015), a wrong interpretation or use of trait associations can lead to erroneous selection decisions or interventions. Therefore, knowledge on network structures among traits might be useful in addition to knowledge on trait correlations with respect to indirect selection efforts.

Genomic correlations, networks, and model performance criteria varied between the Dent and Flint panels, and incorporating genomic networks into prediction models improved goodness of fit more in Dent than in Flint. As the DH lines in the Dent panel were genetically more diverse than in the Flint panel (Lehermeier *et al.* 2014), this could imply that formulating an SEM is more advantageous when dealing with a diverse set of genotypes. We also noted that the DIC in the single-structure SEM was more variable in Flint than in Dent. Variable selection through the network algorithms was more stringent in Flint and, therefore, the SEM differed more from each other and from the MTM than in Dent (Table 2). Based on the comparison of the two data sets, the impact of genetic structure on network inference could not be shown conclusively. In combination with an investigation of methods for control of variable selection intensity (*i.e.*, propensity of an algorithm, test, or score to a sparser network), this topic warrants further research.

In both panels, the marginal logL was higher for many SEM than for MTM, even though the network structure on the covariance matrices implied variable selection. This shows that restrictions on the covariance structures among traits were generally supported by the data. Following the principle of parsimony, a fully recursive structure might not be the best representation of connections among traits.

### Choice of method for network construction

Our results suggest that the choice of method for network learning is crucial, and that a thorough assessment of network structures is necessary when dealing with real-life data. Resulting networks might not explain or fit the data better than a full network as seen here (especially for the genomic component in Flint). Assessment of the different network structures was done by considering several model quality criteria (DIC, logL, number of parameters, and cross-validated predictive ability) together, and by evaluating if these criteria ranked networks consistently. Marginal likelihoods are used routinely to evaluate the plausibility of prior assumptions (*e.g.*, Spiegelhalter *et al.* 2002) from the observed data. After we learned the BN, we translated them into prior assumptions for SEM, and then assessed the various SEM for their priors via marginal likelihoods. DIC conveys information on number of effective parameters in model comparisons. Different prior assumptions translate into different numbers of effective parameters in the SEM, *i.e.*, model complexity varies over networks. As DIC reflects model complexity, it is a natural companion to the marginal likelihood for SEM ranking. While goodness-of-fit criteria evaluate the quality of data description by a network, predictive ability reflects the generalization of structure estimation from one subset of the data (training set) to another subset (test set). However, true structures of traits remain unknown and validating inferred connections in experiments with a broad range of genetic material after hypothesis generation with BN and SEM is crucial.

SEM analysis suggested score-based approaches are better for learning the structure of the genomic networks, and constraint-based approaches are better for the residual networks. This might have resulted from constraint-based algorithms having been more restrictive (given the chosen significance level) and from residual networks being sparser than genomic networks. Evidence for the significance of edges (*i.e.*, support of the edge in >70% of the bootstrap samples) was generally higher in the constraint-based networks than in the score-based networks.

Influence of the significance level in the constraint-based algorithms warrants further investigation. However, it might be expected that a larger *α* for learning BN from the residuals would result in more complex networks, and, at some point, that additional complexity would result in lower predictive ability and/or overfitting. Advanced network-construction algorithms, such as max-min hill-climbing (Tsamardinos *et al.* 2006), combine a constraint-based edge search with a score-based directing of the edges. Such combined approaches should be investigated in future research because they allow variation of the significance level in combination with a score-based network search (*i.e.*, directing arcs).

### Interpretation of network structures

If variables A and B are directly associated with each other, and B and C are alike, then an association between A and C would be a logical consequence, even if there is no direct association between A and C (*cf*. part A of Figure 3). When a BN is inferred, such associations that can be explained by a chain of other associations are not depicted as connections. This means that a connection in a network is more reliable than a significant association between two variables because BN eliminate connections that are unlikely to be direct.

Therefore, BN connections provide information beyond mere pairwise associations between traits. If there were a causal connection between child and parent variable, then the child variable would change with a change of the parent variable. For example, if silking time in Dent material is changed, *e.g.*, by early seeding, it is likely that PH at harvest changes too, according to the residual network in the Dent panel. If the genotypic value of PH is changed in the Dent panel, *e.g.*, by selection, this change is likely to affect the genotypic value of DMY, according to the genomic network in the Dent panel. Nonetheless, the interpretation of BN connections as causal effects is delicate and needs further assumptions than those made here, especially the assumption of absence of additional variables influencing those already included in the study. Thus, we suggest interpretation of our networks as an overall association among more than two entities, *i.e.*, values of the child variable are associated with values of the parent variable.

Residual networks followed some temporal order as traits measured at harvest (PH, DMY, and DMC) depended on traits measured during the vegetation period (DtTAS and DtSILK). Additionally, the inference of direction between DtTAS and DtSILK in the residual networks was relatively uncertain with 52 and 55% of bootstrap samples supporting the direction. This might be because both flowering traits are determined by the time of transition from vegetative to reproductive growth (TVR) and no direct dependence between them truly exists, as both traits depend on TVR. The connection between DtTAS and DtSILK would then be an example of an induced connection by an unobserved confounder in a network.

A conclusion of our study is that by illustrating trait connections with respect to their genomic and residual nature separately and identifying spurious connections, a clearer picture on traits can be gained. This can be useful for multiple-trait prediction and indirect selection.

## Supplementary Material

Supplemental material is available online at www.g3journal.org/lookup/suppl/doi:10.1534/g3.117.044263/-/DC1.
