# Supplementary material for: Bayesian Networks Illustrate Genomic and Residual Trait Connections in Maize (Zea mays L.)
Source: G3 (Bethesda). 2017 Jun 21;7(8):2779–89. doi: 10.1534/g3.117.044263 (PMC5555481; doi:10.1534/g3.117.044263)
Supplement: Supplementary file 1 [file 2779FigureS1.pdf]

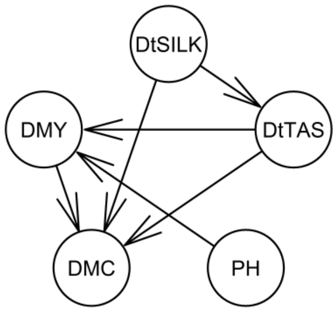

$$\Lambda = \begin{pmatrix} 0 & 0 & 0 & 0 & 0 \\ 0 & 0 & 0 & 0 & 0 \\ \lambda_{DtTAS,DtSILK} & 0 & 0 & 0 & 0 \\ 0 & \lambda_{DMY,PH} & \lambda_{DMY,DtTAS} & 0 & 0 \\ \lambda_{DMC,DtSILK} & 0 & \lambda_{DMC,DtTAS} & \lambda_{DMC,DMY} & 0 \end{pmatrix}$$

coefficients ordered as:
DtSILK – PH – DtTAS – DMY - DMC

Figure S1. Transformation from a DAG to a structure matrix $\mathbf{\Lambda}$. An example of a directed, acyclic graph and its respective structure matrix is shown. The structure was learned by a Bayesian network algorithm.
