# Supplementary material for: Bayesian Networks Illustrate Genomic and Residual Trait Connections in Maize (Zea mays L.)
Source: G3 (Bethesda). 2017 Jun 21;7(8):2779–89. doi: 10.1534/g3.117.044263 (PMC5555481; doi:10.1534/g3.117.044263)
Supplement: Supplementary file 2 [file 2779FigureS2.pdf]

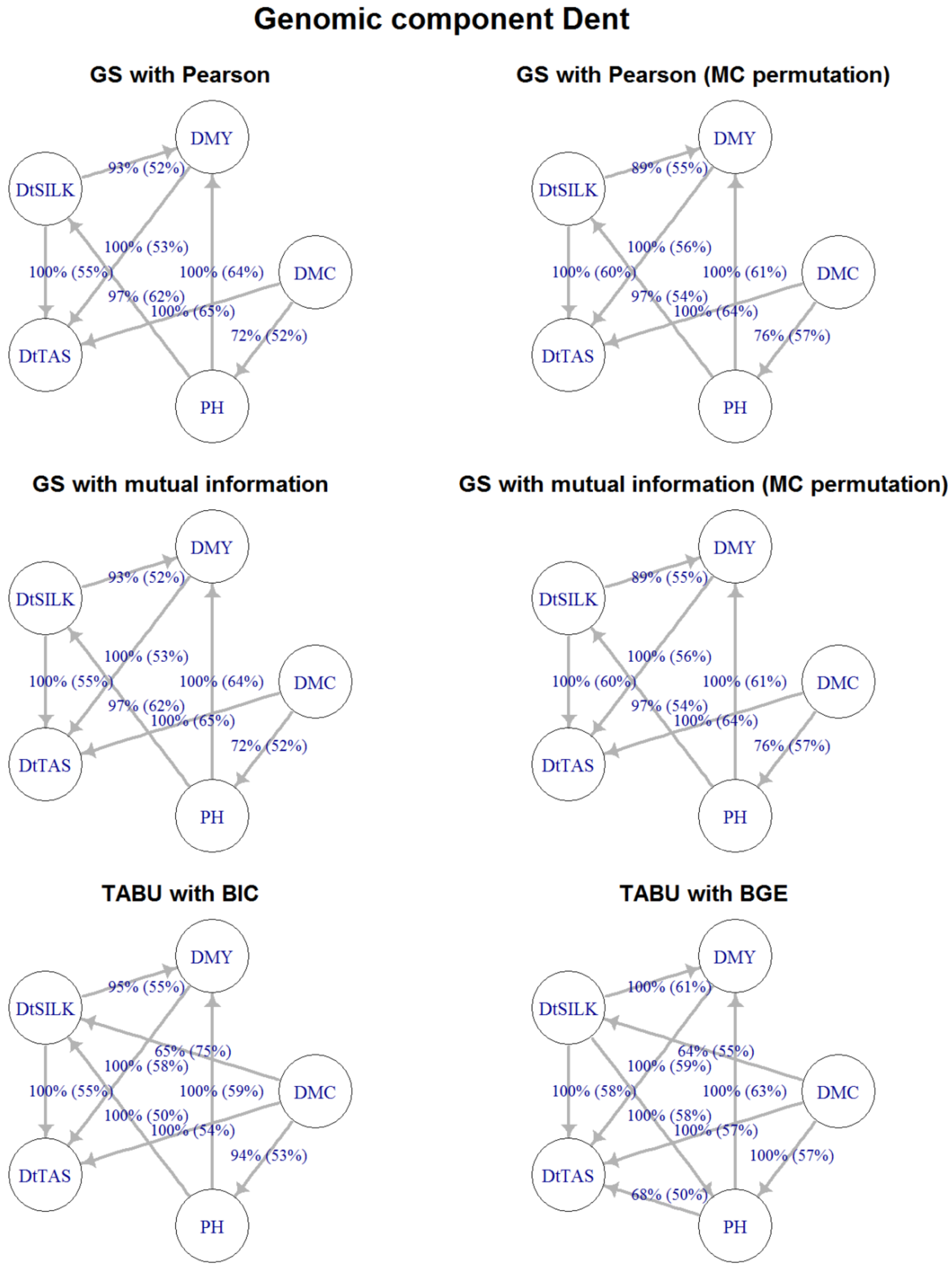

Figure S2. Networks of the genomic component in Dent. All networks identified the connections from DtSILK to DtTAS, from DtSILK to DMY, from DMC to PH, from PH to DMY, from DMY to DtTAS, from DMC to DtTAS, and between PH and DtSILK. Both score-based approaches (TABU 1 and 2) displayed an extra edge from DMC to DtSILK. The score-based approach with the BGE score (TABU 2) showed an additional edge from PH to DtTAS. The SEM favored the tabu-search with the BIC score (TABU 1) over all other settings. Labels of edges indicate the proportion of bootstrap samples supporting the edge and (in parentheses) the proportion having the direction shown. Edges that were not significant in the averaging process due to a network-internal empirical test on the arc’s strength are not shown.
