# Supplementary material for: Bayesian Networks Illustrate Genomic and Residual Trait Connections in Maize (Zea mays L.)
Source: G3 (Bethesda). 2017 Jun 21;7(8):2779–89. doi: 10.1534/g3.117.044263 (PMC5555481; doi:10.1534/g3.117.044263)
Supplement: Supplementary file 3 [file 2779FigureS3.pdf]

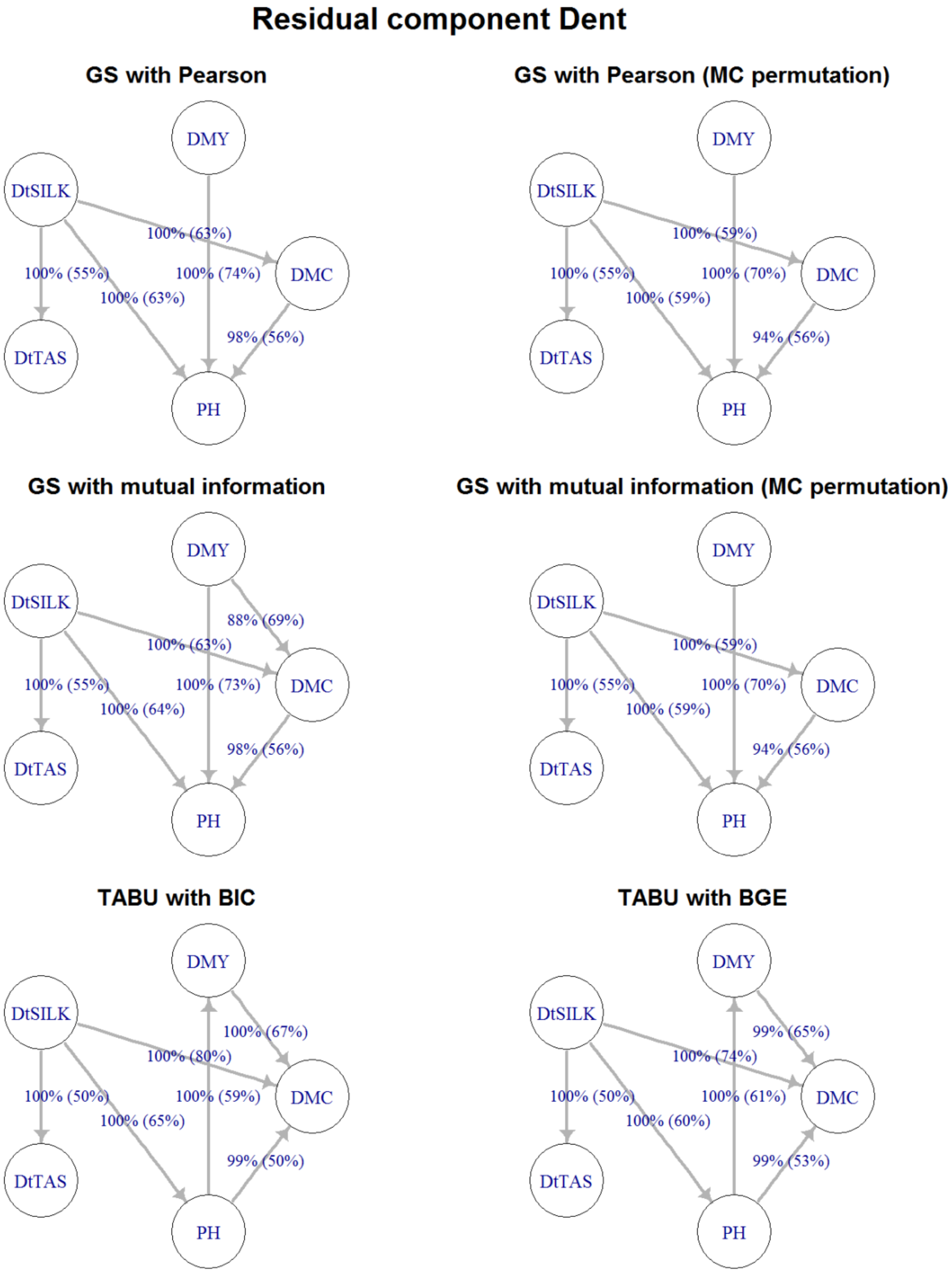

Figure S3. Networks of the residual component in Dent. All algorithms showed edges from DtSILK to DMC, from DtSILK to PH, from DtSILK to DtTAS, between DMY and PH, and between DMC and PH. One constraint-based approach (GS 3) and the score-based approaches (TABU 1, 2) identified an additional connection from DMY to DMC. The SEM favored the Grow-Shrink algorithm with the mutual information criterion (GS 3) over all other settings. Labels of edges indicate the proportion of bootstrap samples supporting the edge and (in parentheses) the proportion having the direction shown. Edges that were not significant in the averaging process due to a network-internal empirical test on the arc's strength are not shown.
