# Supplementary material for: Bayesian Networks Illustrate Genomic and Residual Trait Connections in Maize (Zea mays L.)
Source: G3 (Bethesda). 2017 Jun 21;7(8):2779–89. doi: 10.1534/g3.117.044263 (PMC5555481; doi:10.1534/g3.117.044263)
Supplement: Supplementary file 4 [file 2779FigureS4.pdf]

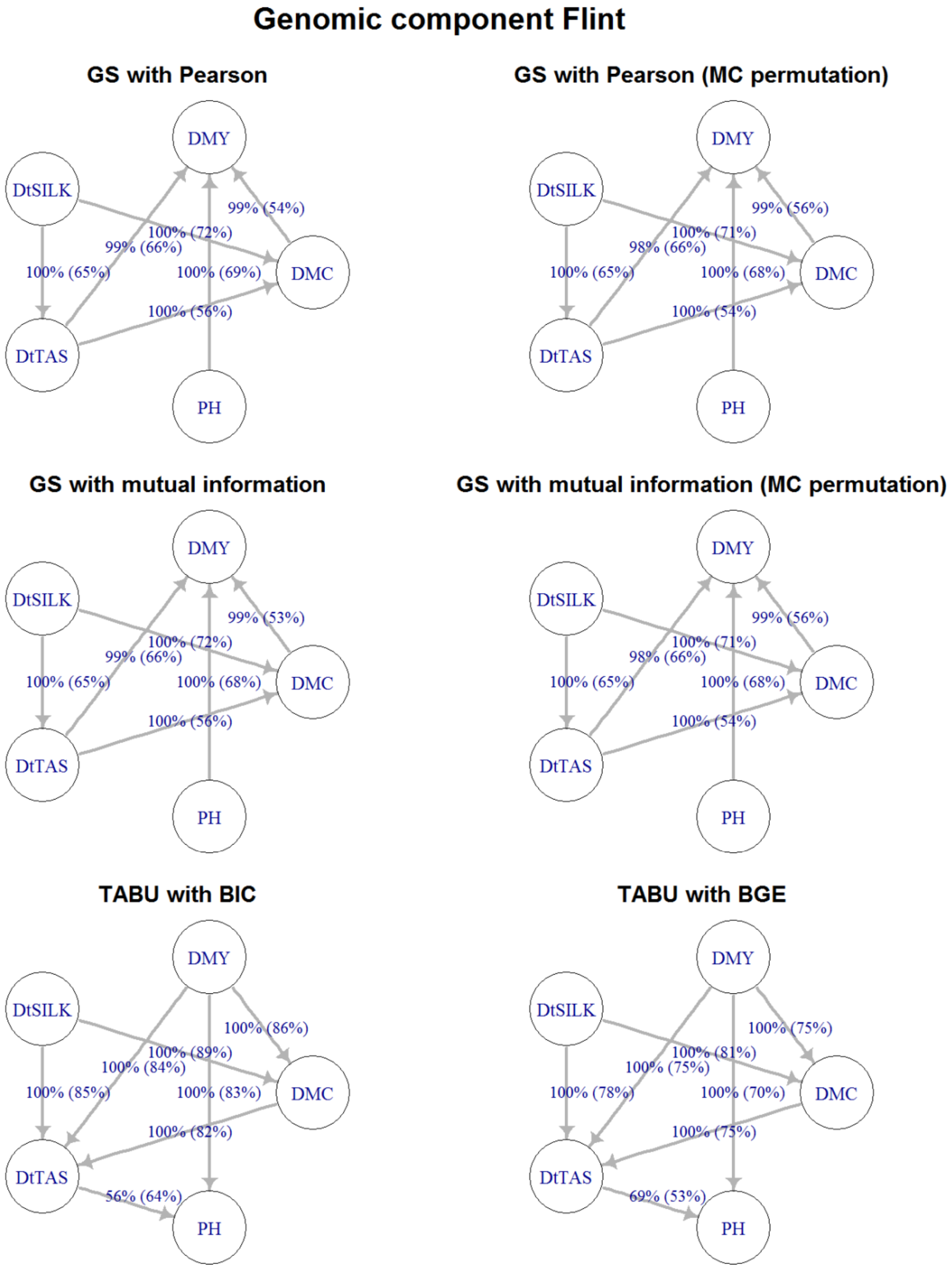

Figure S4. Networks of the genomic component in Flint. All algorithms identified the connections from DtSILK to DtTAS, from DtSILK to DMC, between DMY and DMC, between DMY and PH, between DMY and DtTAS, and between DtTAS and DMC. The score-based approaches (TABU 1, 2) oriented the connections with DMY and between DtTAS and DMC in the opposite direction than the constraint-based approaches (GS 1, 2, 3, 4). They also displayed an additional edge from DtTAS to PH. The SEM favored the tabu-search algorithms (TABU 1, 2) over all other settings. Labels of edges indicate the proportion of bootstrap samples supporting the edge and (in parentheses) the proportion having the direction shown. Edges that were not significant in the averaging process due to a network-internal empirical test on the arc’s strength are not shown.
