# Supplementary material for: Bayesian Networks Illustrate Genomic and Residual Trait Connections in Maize (Zea mays L.)
Source: G3 (Bethesda). 2017 Jun 21;7(8):2779–89. doi: 10.1534/g3.117.044263 (PMC5555481; doi:10.1534/g3.117.044263)
Supplement: Supplementary file 5 [file 2779FigureS5.pdf]

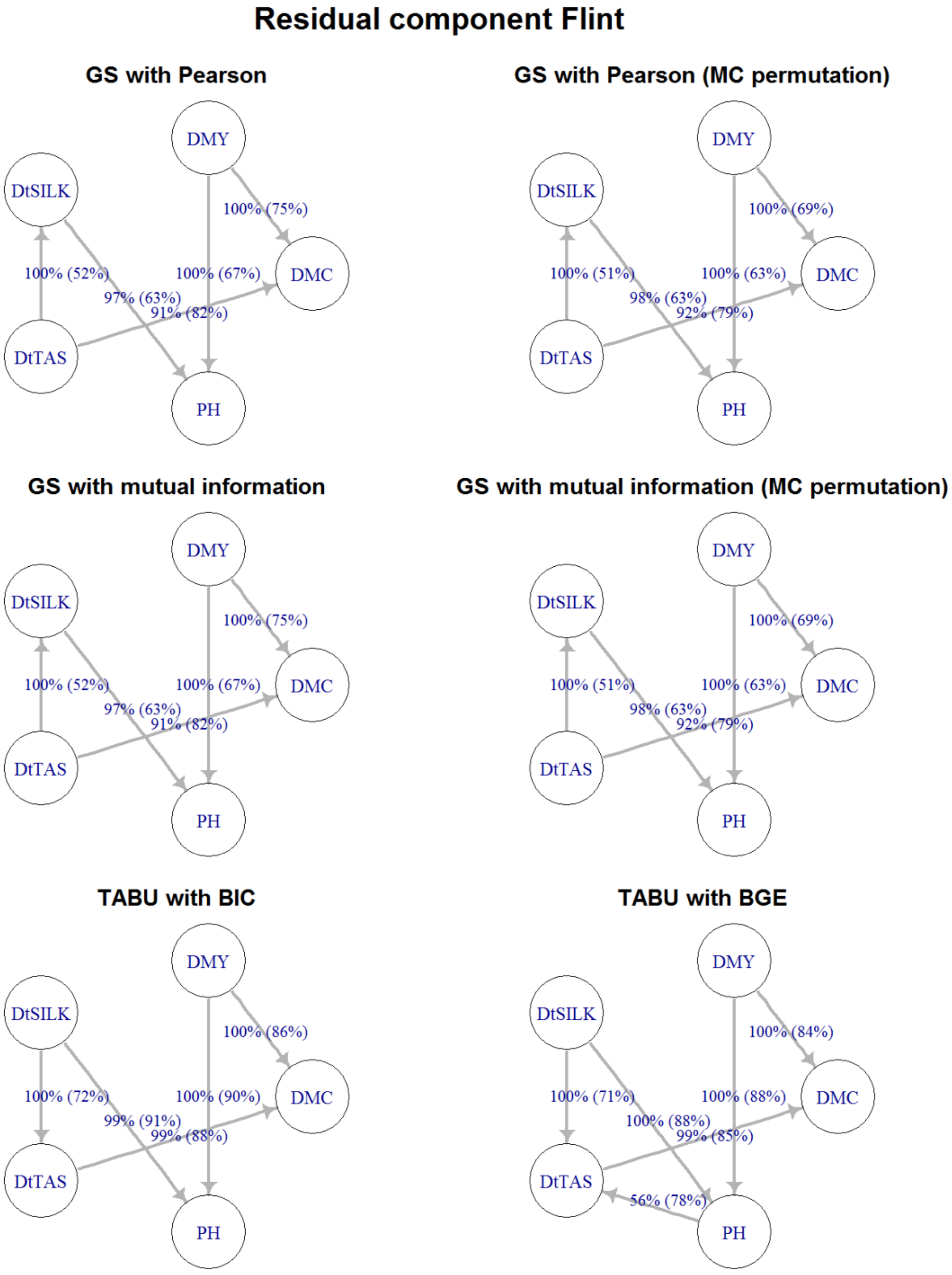

Figure S5. Networks of the residual component in Flint. All algorithms showed edges from DtSILK to PH, from DMY to PH, from DMY to DMC, from DtTAS to DMC, and between DtTAS and DtSILK. The score-based approach with the GPD (TABU 2) identified an additional connection from PH to DtTAS. The SEM favored the Grow-Shrink algorithm with the mutual information criterion (GS 3) over all other settings. Labels of edges indicate the proportion of bootstrap samples supporting the edge and (in parentheses) the proportion having the direction shown. Edges that were not significant in the averaging process due to a network-internal empirical test on the arc’s strength are not shown.
