# Supplementary material for: Bayesian Networks Illustrate Genomic and Residual Trait Connections in Maize (Zea mays L.)
Source: G3 (Bethesda). 2017 Jun 21;7(8):2779–89. doi: 10.1534/g3.117.044263 (PMC5555481; doi:10.1534/g3.117.044263)
Supplement: Supplementary file 7 [file 2779TableS2.pdf]

Table S2. Double-structure evaluation: Predictive abilities[1] with standard deviation for the multiple-trait model (MTM) and for structural equation models (SEM) including both genomic ($\boldsymbol{\Lambda}_{\widehat{U}^*}$) and residual ($\boldsymbol{\Lambda}_{\hat{E}}$) trait structure denoted by the Bayesian network (BN) algorithms they originate from.

BN giving $\boldsymbol{\Lambda}_{\hat{E}}$	BN giving $\boldsymbol{\Lambda}_{\widehat{U}^*}$	DMY[2]	DMC	PH	DtTAS	DtSILK
Dent						
TABU 1	GS 3	0.53 (0.05)	0.64 (0.04)	0.69 (0.04)	0.63 (0.05)	0.68 (0.04)
TABU 2	GS 3	0.53 (0.05)	0.64 (0.04)	0.69 (0.04)	0.63 (0.05)	0.68 (0.04)
TABU 1	GS 1, 2, 4	0.53 (0.05)	0.64 (0.04)	0.69 (0.04)	0.63 (0.05)	0.68 (0.04)
TABU 1	TABU 1, 2	0.53 (0.05)	0.64 (0.04)	0.69 (0.04)	0.63 (0.05)	0.68 (0.04)
TABU 2	TABU 1, 2	0.53 (0.05)	0.64 (0.04)	0.69 (0.04)	0.63 (0.05)	0.68 (0.04)
TABU 2	GS 1, 2, 4	0.53 (0.05)	0.64 (0.04)	0.69 (0.04)	0.62 (0.05)	0.68 (0.04)
-----	-----	0.52 (0.05)	0.64 (0.04)	0.69 (0.04)	0.62 (0.04)	0.68 (0.04)
GS 1, 2, 3, 4	GS 3	0.53 (0.05)	0.63 (0.04)	0.69 (0.04)	0.62 (0.04)	0.67 (0.04)
GS 1, 2, 3, 4	GS 1, 2, 4	0.53 (0.05)	0.63 (0.04)	0.69 (0.04)	0.62 (0.04)	0.67 (0.04)
GS 1, 2, 3, 4	TABU1, 2	0.53 (0.05)	0.63 (0.04)	0.69 (0.04)	0.62 (0.04)	0.67 (0.04)
Flint						
-----	-----	0.64 (0.04)	0.67 (0.05)	0.70 (0.04)	0.74 (0.04)	0.76 (0.04)
TABU 1, 2	TABU 1	0.63 (0.04)	0.67 (0.05)	0.70 (0.04)	0.74 (0.03)	0.76 (0.03)
TABU 1, 2	GS 1, 2, 3, 4	0.63 (0.04)	0.67 (0.05)	0.70 (0.04)	0.74 (0.03)	0.76 (0.03)
TABU 1, 2	TABU 2	0.63 (0.04)	0.67 (0.05)	0.70 (0.05)	0.74 (0.04)	0.76 (0.04)
GS 1, 2, 3, 4	TABU 2	0.64 (0.04)	0.67 (0.05)	0.68 (0.04)	0.73 (0.03)	0.75 (0.03)
GS 1, 2, 3, 4	TABU 1	0.64 (0.04)	0.67 (0.05)	0.68 (0.05)	0.73 (0.04)	0.75 (0.04)
GS 1, 2, 3, 4	GS 1, 2, 3, 4	0.64 (0.04)	0.67 (0.05)	0.68 (0.05)	0.73 (0.04)	0.75 (0.04)

For notation of BN algorithms see material and methods, "Learning genomic and residual Bayesian networks".

[1] Average of 10 random 5-fold cross-validations

[2] Traits: DMY biomass dry matter yield (dt/ha), DMC biomass dry matter content (%), PH plant height (cm), DtTAS days to tasseling (days), DtSILK days to silking (days)
